# Supplementary figures and images for: Genome-Wide Identification and Expression Profiling of Heat Shock Protein 20 Gene Family in Sorbus pohuashanensis (Hance) Hedl under Abiotic Stress
Source: Genes (Basel). 2022 Nov 29;13(12):2241. doi: 10.3390/genes13122241 (PMC9778606; doi:10.3390/genes13122241)

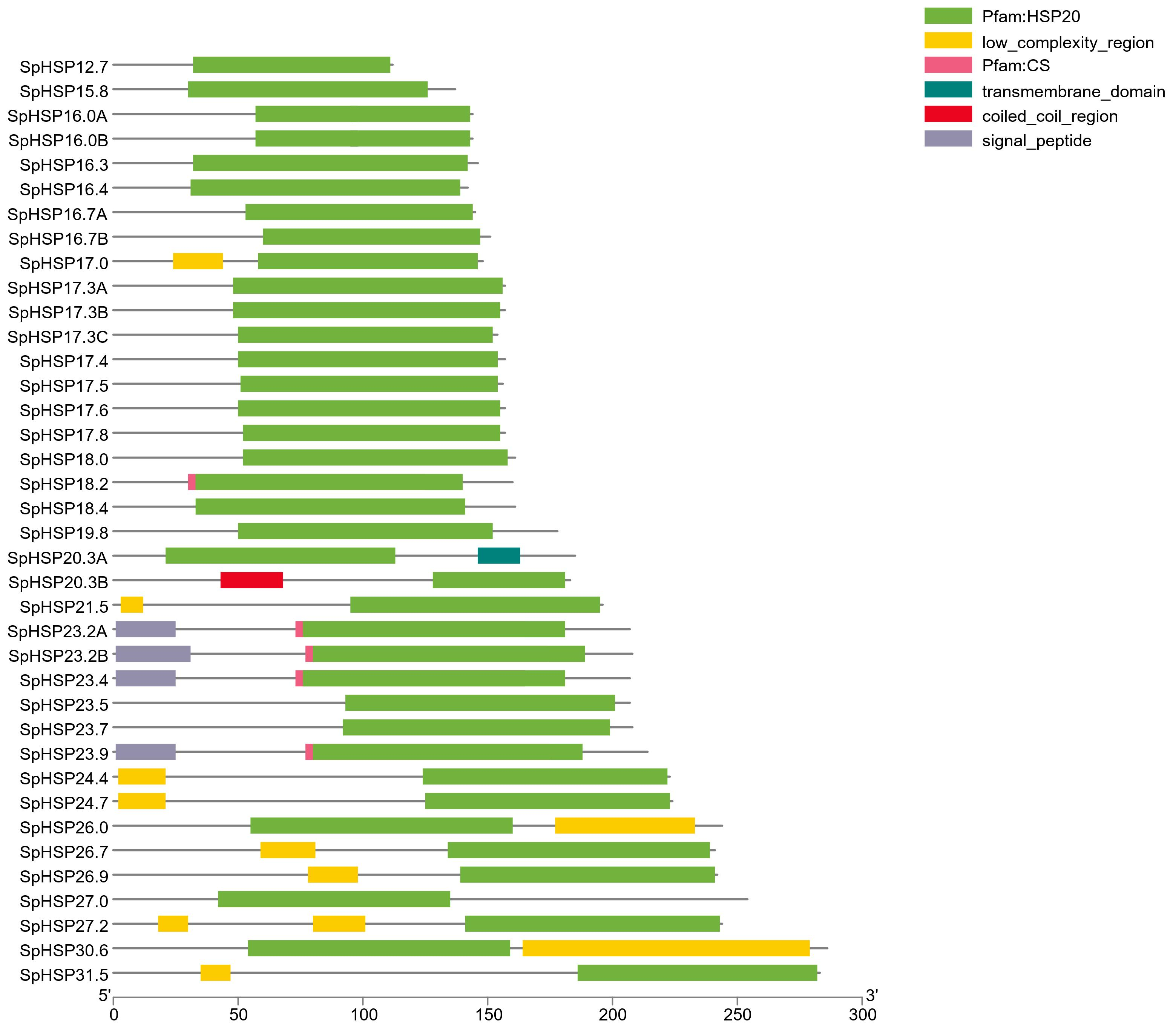

Supplement: Supplementary file 1 [file genes-13-02241-s001.zip › Figure S1.tif]

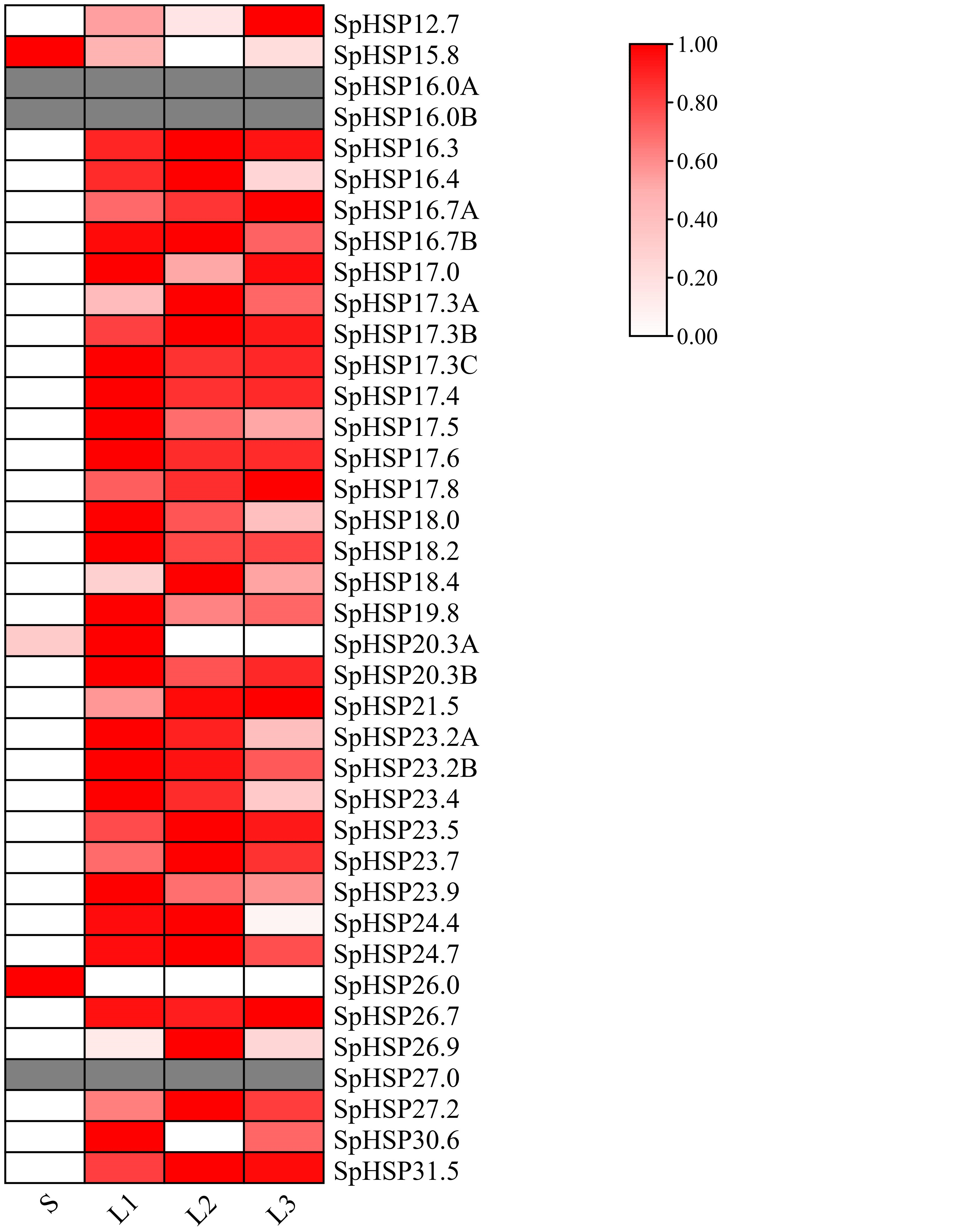

Supplement: Supplementary file 1 [file genes-13-02241-s001.zip › Figure S2.tif]
